# Retrospective comparison of clinical outcomes of robotic-assisted laparoscopic partial nephrectomy through transabdominal or retroperitoneal approaches in patients with T1b renal tumor

**DOI:** 10.1186/s12894-022-01162-w

**Published:** 2022-12-21

**Authors:** Hao Tang, Tianyi Shen, Kai Zhou, Feng Xu, Huichen Lv, Jingping Ge

**Affiliations:** Department of Urology, Jinling Hospital Affiliated to Medical College of Nanjing University, No. 305 East Zhongshan Road, Nanjing, 210002 China

**Keywords:** Renal carcinoma, Robotic-assisted, Laparoscopic partial nephrectomy, Transabdominal approach, Retroperitoneal approach

## Abstract

**Background:**

We compared the intraoperative and postoperative outcomes of robotic-assisted laparoscopic partial nephrectomy (RALPN) via transabdominal or retroperitoneal approaches in patients with stage T1b renal cell carcinoma.

**Methods:**

The medical records for 92 patients who underwent RALPN were retrospectively collected and data on their baseline demographics, duration of operation, duration of renal artery clamping, intraoperative blood loss, recovery time of intestinal functions, surgical margin positive rate, as well as postoperative complications were analyzed.

**Results:**

Of the 92 enrolled patients, 43 and 49 patients were subjected to RALPN via the transabdominal and retroperitoneal approaches, respectively. All patients successfully completed the operation. Baseline characteristics for the transabdominal and retroperitoneal groups were comparable. Differences in operative time, renal artery clamping time, intraoperative blood loss, positive rate of surgical margin, and incidences of postoperative complications between the two approaches were insignificant. The recovery time of intestinal function after operation was significantly shorter in patients subjected to the retroperitoneal approach, relative to those subjected to transabdominal approach (*p* < 0.001).

**Conclusions:**

Application of RALPN via transabdominal or retroperitoneal approaches showed comparable clinical outcomes in patients with stage T1b renal cell carcinoma. The retroperitoneal approach was superior to the transabdominal approach in terms of postoperative intestinal function recovery.

## Background

Renal cell carcinoma is a common urinary system tumor. For patients with clinical stage T1b tumor, partial nephrectomy has the same tumor clearance effect as radical nephrectomy, and can preserve the residual nephron and functions to the greatest extent. Robotic-assisted laparoscopic partial nephrectomy (RALPN) has been shown to markedly reduce the surgical challenges associated with partial nephrectomy and is easier for surgeons to perform [1–5].

Currently, RALPN is performed via transabdominal and retroperitoneal approaches [2–5]. The transabdominal approach is beneficial for incision and suturing during the operation, but it is associated with the risk of damaging the intestines and other abdominal organs. The retroperitoneal approach can avoid the effects of abdominal organ occlusion or abdominal adhesion on the operation, but may lead to peritoneal rupture.

There are no standard recommendations for surgical approaches that are aimed at achieving RALPN [6, 7]. Clinical evidence for efficacies of transabdominal and retroperitoneal approaches are inconclusive [8–11]. A meta-analysis conducted by Cacciamani et al. showed that the retroperitoneal approach led to lower estimated blood loss and shorter operative time than the transabdominal approach [8]. Pavan et al. reported that relative to the transabdominal approach, the retroperitoneal approach is associated with lower estimated blood loss, and shorter operating time as well as length of stay; however, the two approaches exhibit comparable overall and major postoperative complications, warm ischemia time, and positive surgical margins [9]. Hughes-Hallett et al. reported a significant reduction in both estimated blood loss and total operative time in the retroperitoneal group compared with the transabdominal group [11]. Tanaka et al. found comparable operative times, warm ischemic times, and adverse events between the two RALPN approaches [10].

We conducted this retrospective study to compare the intraoperative and postoperative outcomes of RAPLN using transabdominal or retroperitoneal approaches in patients with T1b renal carcinoma.

## Methods

### Study participants

We retrospectively reviewed the medical records of patients with renal carcinoma who underwent RALPN in Department of Urology, Jinling Hospital Affiliated to Medical College of Nanjing University between January 2015 and March 2020.

The inclusion criteria were: patients who were 18 years or older; had a pathological diagnosis of renal carcinoma before operation; had an evaluation of renal function based on serum creatine levels before operation; had creatine levels of 50–110 μmol/L; had no metastasis to other organs; were at stage T1bN0M0; had preoperative assessments of renal tumor based on radius exophytic/endophytic nearness anterior/posterior location (RENAL) and preoperative aspects and dimensions used for anatomic (PADUA) scoring systems [12, 13].

### Procedures

**Fig. 1:**
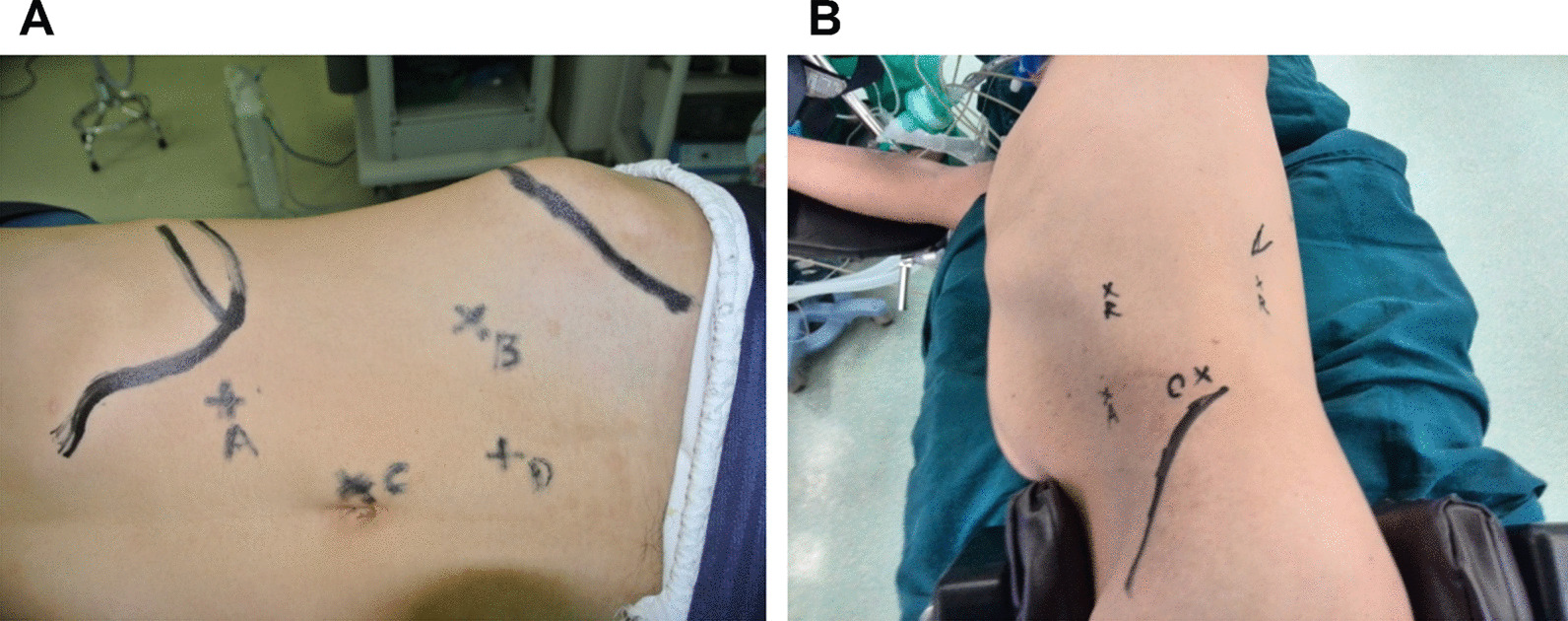
RALPN via transabdominal or retroperitoneal approaches. **A** Transabdominal approach. **B** Retroperitoneal approach

Transabdominal and retroperitoneal RALPN were performed as previously described [11, 14]. A three-arm robotic-assisted surgical system was used for the transabdominal approach (Fig. 1A). Briefly, patients were anesthetized via tracheal intubation and placed in a 45° flank position, with their waists raised. A 12 mm longitudinal skin incision was made 2 cm above the umbilicus as the lens hole and a trocar inserted. After insertion of the lens into the abdominal cavity via the trocar, the pneumoperitoneum pressure was maintained at 12 mmHg. Two 8 mm-trocars were placed at least two transverse fingers below the costal margin of the clavicle midline and above the anterior superior iliac spine, with the lens hole as the center, to form the inverted isosceles triangle of oblique lens hole, as the holes of the head and tail mechanical arm. A 12 mm trocar was obliquely placed below the midpoint of the connecting line between the lens hole and the tail mechanical hole to form an inverted isosceles triangle, as the assistant hole.

The retroperitoneal approach was conducted as follows (Fig. 1B): Patients received general anesthesia via tracheal intubation after which a longitudinal incision of about 12 mm was made over the iliac crest as the lens hole. A trocar was directly inserted into the incision. The lens was inserted into the retroperitoneal space and the pneumoperitoneum pressure maintained at 15 mmHg. An 8 mm trocar was inserted into the anterior and posterior axillary lines of the 12 rib margin under direct vision. A 12 mm trocar was obliquely placed below the midpoint of the line between the lens hole and the anterior axillary mechanical arm hole to form an inverted isosceles triangle.

Selection of the appropriate surgical approach was based on actual growth of the renal tumor, condition of the patient, surgeons’ clinical experience and familiarity with robot operations, and patient decision.

### Assessments

Operative and postoperative follow-up medical records were collected. The operative information included operation time, renal artery clamping time, intraoperative blood loss volume, postoperative intestinal function recovery time, positive rate of surgical margin, and postoperative complications such as infections, bleeding, and urinary leakage. Postoperative follow-up information included routine hematuria, renal function tests, chest X-ray, B-ultrasound and CT. Patients were followed up for 90 days after operation.

### Statistical analysis

The SPSS software v16.0 was used for statistical analyses. Quantitative data are presented as mean ± standard deviation. Comparisons of study groups were performed using the t-test or Fishers’ exact test. *p* ≤ 0.05 was set as the threshold for statistical significance.

## Results

### Baseline characteristics

A total of 92 eligible renal carcinoma patients who underwent RALPN (43 patients subjected to the transabdominal approach and 49 patients subjected to the retroperitoneal approach) were enrolled in this study (Fig. 1). Baseline characteristics for patients who underwent transabdominal RALPN and those who underwent retroperitoneal RALPN were comparable (Table 1). Differences in terms of age (50.5 ± 7.5 vs 52.7 ± 11.8 years, *p* = 0.83), sex (male: 69.8% vs 71.4%, *p* = 0.97), body mass index (20.3 ± 3.78 vs 22.2 ± 3.24 kg/m^2^, *p* = 0.47), tumor diameter (4.9 ± 0.8 vs 5.3 ± 0.6 cm, *p* = 0.54), tumor location (55.8% vs 53.1%, *p* = 0.42), RENAL score (9.2 ± 1.55 vs 8.8 ± 1.62, *p* = 0.14), and PADUA score (9.5 ± 1.90 vs 9.9 ± 1.82, *p* = 0.27) between the two groups were insignificant.

**Table 1:** Baseline characteristics

	Transabdominal approach (n = 43)	Retroperitoneal approach (n = 49)	*p* value
Age (years)	50.5 ± 7.5	52.7 ± 11.8	0.83
Sex			0.97
Male	30 (69.8%)	35 (71.4%)	
Female	13 (30.2%)	14 (28.6%)	
Body mass index (kg/m^2^)	20.3 ± 3.78	22.2 ± 3.24	0.47
Diagnosis			
Renal clear cell carcinoma	43 (100%)	49 (100%)	–
Tumor diameter (cm)	4.9 ± 0.8	5.3 ± 0.6	0.54
Tumor location			0.42
Left	24 (55.8%)	26 (53.1%)	
Right	19 (44.2%)	23 (46.9%)	
RENAL score	9.2 ± 1.55	8.8 ± 1.62	0.14
PADUA score	9.5 ± 1.90	9.9 ± 1.82	0.27

Data are n (%) or mean ± SD

*RENAL* radius exophytic/endophytic nearness anterior/posterior location, *PADUA* preoperative aspects and dimensions used for anatomic

### Comparisons of operation outcomes between the transabdominal and retroperitoneal groups

The 92 patients in both groups successfully completed nephrectomy. During the operation, differences in intraoperative parameters, including duration of operation (transabdominal vs retroperitoneal: 81.2 ± 8.7 vs 70.2 ± 7.5 min, *p* = 0.49), duration of renal artery clamping (16.9 ± 4.7 vs 17.1 ± 4.2 min, *p* = 0.25), and intraoperative blood loss (50.2 ± 7.5 vs 42.8 ± 6.6 mL, *p* = 0.39; Table 2) between the two groups were not significant.

**Table 2:** Comparisons of intraoperative and postoperative outcomes of transabdominal and retroperitoneal approaches

	Transabdominal approach (n = 43)	Retroperitoneal approach (n = 49)	*p* value
Intraoperative parameters			
Duration of operation (minutes)	81.2 ± 8.7	70.2 ± 7.5	0.49
Renal artery clamping time (minutes)	16.9 ± 4.7	17.1 ± 4.2	0.25
Intraoperative blood loss (mL)	50.2 ± 7.5	42.8 ± 6.6	0.39
Postoperative parameters			
Recovery time of intestinal function (day)	5.2 ± 0.5	2.2 ± 0.3	< 0.001
Margin positive	0 (0%)	0 (0%)	–
Postoperative complications	1 (2.3%)	1 (2.0%)	0.87

Data are n (%) or mean ± SD

Regarding postoperative parameters, the recovery time of intestinal functions in the retroperitoneal approach group (2.2 ± 0.3 days) was significantly shorter than that of the transabdominal approach group (5.2 ± 0.5 days, *p* < 0.001). Postoperative histopathological assessment showed that no patient in both groups were surgical margin-positive. Postoperative complications occurred in two patients: one patient with perirenal hematoma in the transabdominal group was cured after conservative treatment while and one patient with urinary leakage in the retroperitoneal group recovered after double J tube drainage.

## Discussion

Nephron-sparing surgery is the gold standard for treating renal cancer with small masses. Minimally invasive procedures with the help of robot are increasingly being used in this patient population. In this retrospective study, we compared the intraoperative and postoperative outcomes of RAPLN using transabdominal or retroperitoneal approaches in patients with T1b renal carcinoma. Our results showed that the transabdominal and retroperitoneal approaches had no significant differences on most intraoperative outcomes, and the retroperitoneal approach was more conducive to the recovery of intestinal function than the transabdominal approach.

Reported evidences of clinical outcomes between transabdominal and retroperitoneal approaches are conflicting. In two previous meta-analysis studies, intraoperative blood loss and duration of operation were significantly low for the retroperitoneal approach, relative to the transabdominal approach; however, differences of postoperative complications between the two robotic approaches were insignificant [8, 9]. Our study supported the generally equivalence clinical outcomes of the transabdominal and retroperitoneal approaches; differences in operation time, renal artery clamping time, blood loss volume, and postoperative complications between patients underwent transabdominal and retroperitoneal approaches were not significant, which were in tandem with findings from several previous studies [10, 11]. Inconsistencies in findings of the above-mentioned studies could be attributed to different study populations, patient conditions, and surgical techniques. More studies should be performed to assess the advantages and disadvantages of the two RALPN approaches, so as to guide clinical practice.

Our study revealed that the retroperitoneal approach was associated with more rapid return of postoperative intestinal function compared with transabdominal approach. This data was consistent with previous study showing shorter recovery time of intestinal function for retroperitoneal approach [15]. We attributed this outcome to the reason that the transabdominal approach provides a larger working space but requires mobilization of the abdominal viscera, especially the intestinal tract, to expose the kidney, which inevitably impaired the recovery of intestinal tract function.

Postoperative pathological examination did not reveal any tumor cells in the surgical margin of all patients from both study groups, indicating that the two RALPN approaches were sufficient for removing all positive tumor lesions. This result was also consistent with that from other studies which demonstrated that no differences were found regarding positive surgical margins between transabdominal and retroperitoneal approaches [9, 16, 17].

We used the RENAL and PADUA scoring systems for preoperative evaluation of renal tumors. The two scoring systems were used to analyze the imaging data of patients before operation, and for comprehensive analysis of scores for tumor sizes, location, tumor invasion depth, and other anatomical characteristics, so as to estimate the feasibility of partial nephrectomy, the difficulty of operation, and the probability of postoperative complications. Results showed that the baseline preoperative RENAL and PADUA scores were comparable between patients that had been subjected to transabdominal and retroperitoneal approaches, which further enhances the trustworthiness and reliability of our findings.

One limitation of this study is the small sample size which may introduce bias into the study results. This is because RALPN has not been fully covered by medical insurance in China and the cost is high, not all partial nephrectomy is assisted by robots and RALPN is lack of popularity in China. However, due to advances in robots, RALPN may reduce the surgical variations that are associated with surgeons' operating skills [18]; and in this study, all surgical procedures were performed by the same surgical team, which further minimized the potential bias of the operations. Another limitation was the lack of the long-term follow-up results of these patients. Therefore, further studies in larger cohort with longer follow-up results are warranted.

## Conclusions

Performing RALPN via the retroperitoneal or transabdominal approaches were associated with comparable surgical outcomes, and the retroperitoneal approach was superior to the transabdominal approach in terms of postoperative intestinal function recovery. Our findings provide a basis for further studies on transabdominal or retroperitoneal approaches for RALPN.

## Data Availability

The data that support the findings of this study are available from the corresponding author upon reasonable request.

## Abbreviations

RALPN: Robotic-assisted laparoscopic partial nephrectomy
CT: Computed tomography
MRI: Magnetic resonance imaging
RENAL: Radius exophytic/endophytic nearness anterior/posterior location
PADUA: Preoperative aspects and dimensions used for anatomic
